# Biochar Mediated-Alleviation of Chromium Stress and Growth Improvement of Different Maize Cultivars in Tannery Polluted Soils

**DOI:** 10.3390/ijerph18094461

**Published:** 2021-04-22

**Authors:** Muhammad Asaad Bashir, Xiukang Wang, Muhammad Naveed, Adnan Mustafa, Sobia Ashraf, Tayyaba Samreen, Sajid Mahmood Nadeem, Moazzam Jamil

**Affiliations:** 1 Department of Soil Science, Faculty of Agriculture and Environment, The Islamia University of Bahawalpur, Bahawalpur 63100, Pakistan; m.asaadbashir@gmail.com (M.A.B.); moazzam.jamil@iub.edu.pk (M.J.); 2 College of Life Sciences, Yan’an University, Yan’an 716000, China; 3 Institute of Soil and Environmental Sciences, University of Agriculture, Faisalabad 38040, Pakistan; sobiaashraf13@googlemail.com (S.A.); tybasamreen@gmail.com (T.S.); 4 Biology Centre, SOWA RI, Czech Academy of Sciences, 37005 Ceske Budejovice, Czech Republic; adnanmustafa780@gmail.com or; 5 Burewala Sub-Campus, University of Agriculture Faisalabad, Vehari 61100, Pakistan; sajidmn92@hotmail.com

**Keywords:** chromium toxicity, biochar, maize, tannery polluted soils, antioxidant enzymes, Kasur and Sialkot, tannery polluted soils

## Abstract

Soil pollution with heavy metal is a serious problem across the globe and is on the rise due to the current intensification of chemical industry. The leather industry is one of them, discharging chromium (Cr) in huge quantities during the process of leather tanning and polluting the nearby land and water resources, resulting in deterioration of plant growth. In this study, the effects of biochar application at the rate of 3% were studied on four maize cultivars, namely NK-8441, P-1543, NK-8711, and FH-985, grown in two different tannery polluted Kasur (K) and Sialkot (S) soils. Maize plants were harvested at vegetative growth and results showed that Cr toxicity adversely not only affected their growth, physiology, and biochemistry, but also accumulated in their tissues. However, the level of Cr toxicity, accumulation, and its influence on maize cultivars varied greatly in both soils. In this pot experiment, biochar application played a crucial role in lessening the Cr toxicity level, resulting in significant increase in plant height, biomass (fresh and dry), leaf area, chlorophyll pigments, photosynthesis, and relative water content (RWC) over treatment set as a control. However, applied biochar significantly decreased the electrolyte leakage (EL), antioxidant enzymes, lipid peroxidation, proline content, soluble sugars, and available fraction of Cr in soil as well as Cr (VI and III) concentration in root and shoot tissues of maize plant. In addition to this, maize cultivar differences were also found in relation to their tolerance to Cr toxicity and cultivar P-1543 performed better over other cultivars in both soils. In conclusion, biochar application in tannery polluted soils could be an efficient ecofriendly approach to reduce the Cr toxicity and to promote plant health and growth.

## 1. Introduction

Soil contamination with heavy metals is an increasingly prominent issue worldwide and is growing due to the use of heavy metal-loaded materials in industries. The untreated waste products of industries contain high concentrations of toxic heavy metal, and thus, induce environmental health risks due to their persistence in nature and tendency to accumulate in food; hence, they cause severe human health hazards [1]. A significant amount of industrial effluent loaded with heavy metal is being discharged into the environment, resulting in increased agricultural soil contamination annually [2,3]. In Pakistan, the leather tanning processes are the largest contributors of soil contamination with chromium (Cr) [4,5,6]. More than 800 tanning units are present on eastern fringe (Kasur and Sialkot) of Pakistan due to the abundance of water, easily available labor, and inexpensive cost of materials [7]. However, improper waste management of this industry and discharge of effluent without any treatment is a major cause of soil contamination [8].

Chromium is the most commonly used heavy metal in the leather industry during tanning processes [9] and is usually discharged with wastewater by repeated (3–4 times) tanning sessions [10]. Chromium, even at low concentrations, is toxic to plants as well as animals [11,12,13]. Cr toxicity evokes considerable stress symptoms in plants, i.e., alterations in the germination process, reduced photosynthesis, and disturbance in nutrient uptake, which affects plant growth, crop yield, and total dry matter production [12,14,15]. Chromium also results in harmful effects of osmotic balance and metabolic activities by damaging the plant cell membrane that leads to electrolyte leakage (EL) and lipid peroxidation [16]. Moreover, Cr toxicity induces oxidative stress in plants by forming reactive oxygen species (ROS) that damages the cell molecules and impairs the antioxidant system [17,18]. Through the food chain, acute exposure of Cr in humans causes severe health problems, such as gastrointestinal, respiratory, cardiovascular, neurological, immunological, reproductive, and developmental problems [4,19]. In addition, a more destructive effect of Cr is the induction of various types of cancers, including lung, skin, kidney, and bladder [20,21,22].

It should be acknowledged that the toxicity of Cr depends upon its valence state. Naturally, Cr is found in multiple oxidation states in the range of −2 to +6 [23,24], but Cr (VI) and Cr (III) dominate in the environment [25,26]. Chromium (III) is relatively harmless, less soluble/mobile, and naturally present in stable form [27,28]. Conversely, Cr (VI) is considered to be highly toxic, soluble/mobile, and causes severe health risks [29,30]. Chromium (VI) rarely occurs in nature and is usually the product of anthropogenic activities [31]. In the tannery industry, predominantly Cr (III) is released through wastewater effluent [9,15]. Here, redox reaction performs very important role in the interconversion of most dominant species of Cr, i.e., Cr (III) and Cr (VI) [30]. Toxicity caused by the presence of large amount of Cr in tannery waste polluted soils could consequence in the absence of vegetation cover [32]. Therefore, organic amendments (manure, biosolids, compost, biochar, etc.) are generally used to enhance soil health and crop growth due to their valuable effects on soil fertility by enhancing soil aggregation, soil aggregate stability, and other physico-chemical characteristics of soil [33,34,35].

Biochar is a recalcitrant fine-grained porous organic substance produced by pyrolysis of biomass in oxygen limited conditions [32,36]. It improves soil organic matter (SOM) and water holding capacity, reduces the nutrient loss, plays an important role in metal sorption [37,38,39,40] including heavy metals and stimulates the microbial activity of soil [32,41]. It has comparatively extensive surface area with high degree of porosity and structured carbon matrix [42] which acts as a pollutant sorbent [43]. It can make precipitates of Cr by reacting with various mineral components (oxidates, carbonates, or phosphates) [44,45]. Numerous oxygen containing acidic (carbonyl, carboxylic, hydroxyl, lactone, phenol, etc.) and basic (chromene, ketone, pyrone, etc.) functional groups may also be present in biochar, which binds metals through complexation [39,46,47]. Moreover, biochar could enhance supply of proton to reduce Cr (VI) into its less toxic Cr (III) form [47,48,49]. Many researchers have previously described the biochar potential to improve the soil characteristics and nutrient uptake by plants, especially under pollutant stress conditions [50,51,52]. Only a limited literature is available on physiological as well as biochemical responses of maize cultivars to biochar applied in soil polluted with indigenous tannery waste [12,47,53].

Therefore, we hypothesized that biochar may reduce the toxicity of Cr (VI) by reducing it into its less toxic form, i.e., Cr (III) and decrease its accumulation in plants. Hence, keeping in view the impact of biochar application in promoting soil characteristics along with the removal of toxic pollutants, a pot study was designed to assess the Cr availability and potential transfer to maize cultivars in tannery polluted soils collected from two districts (Kasur and Sialkot) of Punjab, Pakistan.

## 2. Materials and Methods

### 2.1. Production of Biochar and Its Characterization

Air and oven dried (50 °C) sugarcane bagasse (SB) with 10–15% moisture content was used as feedstock to produce biochar. The increase in muffle furnace temperature was 8–10 °C min^−1^, which was maintained at a residence time of 20 min on attaining 350 °C temperature [54].

The physical parameters of SB biochar were measured by using a suspension ratio of 1:20 (*w*/*v*) prepared in deionized water after shaking on a mechanical shaker for one and a half hours [55].

The ammonium acetate compulsory displacement method [56], with little modification, was used to determine cation exchange capacity (CEC) of biochar (Table 1).

The moisture content (%) of biochar was measured through the difference method by calculating the fresh and dry (dried in an oven at 65 °C) weight:

$$Moisture\;content\;\left(\%\right)\;=\frac{Fresh\;weight-Dry\;weight}{Dry\;weight}\times 100$$

The volatile matter content (%) of biochar was calculated as the weight loss after combustion in a ceramic crucible with a loose ceramic cap at 850–900 °C (6 min).

The ash content (%) of biochar was estimated by the heating of biochar in a muffle furnace at 200 °C for 1 h and then at 750 °C for an additional 4 h with no ceramic cap [57]:

$$Ash\;content\;\left(\%\right)\;=\frac{Weight\;of\;ash}{Weight\;of\;biochar}\times 100$$

The fixed carbon content (%) was measured by difference method after measuring the moisture, volatile matter, and ash content [46]:

$$Fixed\;carbon\;\left(\%\right)\;=100\;-\left(Moisture+Volatile\;matter+Ash\right)\;\%$$

The conversion efficiency (yield) of biochar was measured by using the following equation:

$$Conversion\;efficiency\;\left(\%\right)\;=\frac{Weight\;of\;biochar\;}{Weight\;of\;feedstock}\times 100$$

A surface area analyzer (NOVA 1200; Quantachrome Instruments, Boynton Beach, FL, USA) was used for determining the surface area and pore volume of biochar according to Brunauer–Emmett–Teller (BET) (EMSL Analytical, New York, NY, USA) nitrogen adsorption method at 77 Kelvin [58].

The detection of major elements, i.e., carbon (C), hydrogen (H), and nitrogen (N) was carried out at high-temperature catalyzed combustion through a CHN elemental analyzer (Carlo-Erba NA-1500) (SPW Industrial, Laguna Hills, CA, USA) and resulting CO_2_, H_2,_ and NO_2_ gases were detected by using infrared technique.

Difference method was used for the determination of oxygen (O):

$$Oxygen\;\left(\%\right)\;=100-\left(Ash+carbon+hydrogen+nitrogen\right)\;\%$$

The dry-ashing method with little modification was used to measure macronutrients, i.e., phosphorus (P), potassium (K), calcium (Ca), and magnesium (Mg) as well as micronutrients, i.e., iron (Fe), zinc (Zn), copper (Cu), and manganese (Mn) in biochar samples [59]. Therefore, biochar samples were ashed for 8 h in a muffle furnace (Gallonhop, England, UK) and then HNO_3_ (5 mL) was added to each vessel and heated (120 °C) on a hot plate until dry. Upon cooling, HNO_3_ (1 mL) and H_2_O_2_ (4 mL) was added, and samples were again heated (120 °C) by placing them back on a preheated plate, until dry, and then solubilized and filtered. Inductively coupled plasma with optical emission spectroscopy (ICP–OES) (Perkin Elmer Optima 2100 DV) (PerkinElmer, Massachusetts, USA) was used for nutrient analysis.

### 2.2. Collection and Analysis of Tannery Polluted Soil Samples

Tannery polluted soil samples were collected from the vicinity of tanning industry of two districts of Punjab, Kasur (K) and Sialkot (S). These soil samples after sieving were subjected to various physico-chemical analysis (Table 2). Electrical conductivity (EC) meter was used to measure EC of the extract of the saturated soil paste. The texture of soil was determined by the hydrometer method [60]. Soil organic carbon was estimated by a TRL-TOC model analyzer (TRL Instruments^®^ 1328. Cadde, Demirag Apt. No: 14/5, Ankara, Turkey). Soil CEC was determined by following the method of Sumner et al. [61] and calcium carbonate was measured by following the method of Leoppert et al. [62]. Soil total N was determined by the Kjeldahl method [63], available P with sodium bicarbonate [64], and extractable K by the method detailed in Richards [65]. Atomic adsorption spectrophotometer (Perkin Elmer Aanalyst-100, USA) was used for the total Cr concentration (Cr III and VI) after aqua regia ((HCl:HNO_3_ = 3:1) digestion of the soil samples [66]. The chromium (VI) concentration in soil was measured by using 1,5-diphenylcarbazide following the DTPA method [67] with modifications [68] at 540 nm wavelength using spectrophotometer (Shimadzu UV-1800) and Cr (III) was determined by subtracting the concentrations of Cr (VI) from total Cr.

### 2.3. Experimental Design and Setup

A pot experiment, comprising 16 different treatments in triplicates by applying SB biochar at a rate of 3% (*w*/*w*) in the tannery polluted soils of K and S, using four different maize cultivars (NK-8441, P-1543, NK-8711, and FH-985) was performed. The arrangement of the experiment was according to a completely randomized design (CRD). Pots were filled with sieved soil (6 kg) and the recommended dose of N (180 Kg ha^−1^), P (120 Kg ha^−1^), and K (90 Kg ha^−1^) fertilizers was also applied to fulfill the initial nutrient requirements of maize plants by using urea, diammonium phosphate, and sulfate of potash, respectively. The arrangement of the experiment was with three replicates. Five seeds of selected maize cultivars were sown, and seven days after emergence, thinning was done to two plants per pots.

### 2.4. Measuring Growth Parameters

At vegetative growth (45 days), plants were harvested and roots/shoots length of maize plant was recorded by using meter rod. Plant fresh and dry weight (after oven drying at 65 °C for 72 h) of maize root/shoot part was determined by using a weighing balance. To calculate the leaf area (Y) of maize cultivars, the following equation was used, which was earlier described by Chanda and Singh [69]:

$$Leaf\;area\;\left(Y\right)\;=Length\times Width\times 0.75$$

### 2.5. Measuring Maize Physiological Characteristics

#### 2.5.1. Physiological Parameters

For photosynthetic pigments and gaseous exchange measurements, the fully matured top second maize leaf from each treatment was collected. SPAD (soil plant analysis development) chlorophyll content was measured with the help of SPAD-502 m (Konica-Minolta, Tokyo, Japan). Chlorophyll (a and b) and total carotenoid contents were also analyzed by the weighing of fresh leaves (0.5 g) and homogenized with 80% acetone (10 mL). The extracts were centrifuged (10,000 rpm for 15 min at 4 °C) and absorbance of the supernatant was measured at 645, 663, and 480 nm by using a spectrophotometer (Shimadzu UV-1800) [70].

The CIRAS-3 portable photosynthesis system was used for gaseous exchange measurements, such as transpiration rate (E), plant photosynthetic rate (A), stomatal conductance (gs), etc. (portable photosynthesis system, Amesbury, MA, USA).

#### 2.5.2. Water Relations of Plants

Water relations of maize plants in terms of relative water contents (RWC) and electrolyte leakage (EL) were determined. The RWC of plant leaf (1 cm^2^) was determined by using the following formula [71]:

$$Relative\;water\;content\;\left(\%\right)\;=\frac{Fresh\;Weight-Dry\;Weight}{Turgid\;Weight-Dry\;Weight}\times 100$$

The turgid weight was determined by maintaining the leaf in a humid environment (4 °C for 24 h).

Electrolyte leakage was measured by following the method of Lutts et al. [72] with little modification. Leaf sample (1 cm^2^) were placed in test tubes with distilled water (10 mL), and electrical conductivity, *EC*_1_, was measured with an EC meter at room temperature. The tubes were placed on a mechanical shaker for 2 h in water and the *EC*_2_ was calculated. The tubes were then autoclaved (120 °C) and *EC*_3_ was determined upon cooling. Electrolyte leakage was measured by using the following formula:

$$Electrolyte\;Leakage\;\left(\%\right)\;=\frac{EC_{2}-EC_{1}}{EC_{3}}\times 100$$

### 2.6. Measuring Maize Biochemical Attributes

#### 2.6.1. Determination of Stress-Related Metabolites

Stress-related metabolites, i.e., total soluble sugars concentrations, were analyzed by extracting the plant sample (0.1 g) in ethanol solution (80%). The extracted material was taken in test tubes (25 mL) and added 6 mL anthrone reagent (150 µg of anthrone in 72% H_2_SO_4_) to each tube, heated in a boiling water bath (10 min), ice cooled, and incubated at room temperature (25 °C) for 20 min. Spectrophotometer was used for measuring optical density at 625 nm and standard curve prepared from glucose was applied for the calculation of soluble sugars [73].

For proline content, the leaf sample (1 g) was homogenized in sulphosalicylic acid (3%), and filtered and the mixture was heated in a test tube on addition of glacial acetic acid and acid ninhydrin in a water bath (100 °C for one hour). The tubes were ice cooled to stop the reaction, extracted with toluene, and spectrophotometer absorbance at 520 nm was recorded [74].

For lipid peroxidation, malondialdehyde (MDA) content was determined by homogenizing the plant fresh leaf (200 g) in trichloroacetic acid (0.1%) and centrifuged (10,000 rpm for 15 min). The supernatant (1 mL) was mixed with 20% trichloroacetic acid (2 mL) and 0.5% thiobarbituric acid (2 mL) in test tubes, heated in a water bath (90 °C for half an hour) and ice cooled to stop the reaction. The concentration of MDA content in term of lipid peroxidation was determined by spectrophotometric absorbance at 532 nm [72].

#### 2.6.2. Antioxidant Enzymes Assay

Frozen plant leaf was homogenized in an ice-cold mixture of potassium phosphate buffer (0.2 M) and EDTA (0.1 mM) solution.

Ascorbate peroxidase (APX) activity was measured by a decrease in the spectrophotometer absorbance at 290 nm due to oxidation of ascorbate in the reaction [75].

Glutathione peroxidase (GPX) activity was calculated by a reaction of a sodium azide, glutathione, and GPX solution into a ß-NADPH (nicotinamide adenine dinucleotide phosphate) flask, and the spectrophotometric absorbance at 340 nm was recorded [76].

Catalase (CAT) activity was observed by a decrease in the spectrophotometer absorbance at 240 nm due to loss of H_2_O_2_ [77].

Superoxide dismutase (SOD) activity was determined by monitoring the reaction mixture (sodium phosphate, EDTA, and pyrogallol) at 420 nm on a spectrophotometer [78].

### 2.7. Chromium Speciation in Plant Tissues

The double dry ashing method was used for digestion of plant root and shoot tissues for Cr determination [79]. Cr (VI) in plant tissues was analyzed by following the 1,5-diphenylcarbazide (DPC) method [67] with little modifications [68] using a spectrophotometer (Shimadzu UV-1800) at a 540 nm wavelength. For the total Cr concentration, an atomic absorption spectrophotometer (Perkin Elmer Aanalyst-100) was used and the Cr (III) concentration was determined by the difference method between Cr (VI) and total Cr concentration.

### 2.8. Statistical Data Analysis

All the collected data were analyzed by applying an ANOVA (analysis of variance) test to assess the variation among the mean values of different treatments applied on maize cultivars. Subsequently, Tukey’s HSD (honestly significant difference) test was used to compare the treatment mean values at 5% probability level [80] by using Statistix (version 8.1) (Analytical software, 2005, Florida, USA). Spearman’s correlation analysis, using the program *RStudio* (R Software^®^ version 4.0.2) (R Core Team, Boston, MA, USA), was applied to determine the correlation among all the studied parameters. Moreover, different experimental treatments with respect to all the studied morphological, physiological, and biochemical attributes of different maize cultivars in both K and S soils was also performed to compare the principal component analyses (PCA).

## 3. Results

### 3.1. Impact of Biochar on Maize Growth

In the present study, Cr toxicity adversely affected the plant growth of maize cultivars (NK-8441, P-1543, NK-8711, and FH-985) in tannery waste polluted soils, as shown in Table 3. In the control treatment (without biochar), Cr toxicity intensely reduced the plant height (14.4 cm), root length (6.2 cm), fresh weight of shoot (2.9 g), and root (2.5 g) along with shoot (0.52 g) and root (0.34 g) dry weight, as well as leaf area (20 cm^2^) of maize cultivar FH-985 in S soil. Conversely, biochar (3%) application significantly improved the growth of all the studied maize cultivars in both K and S soils polluted with tannery waste. Here, the highest increase in the plant height (20.2%), root length (21.9%), and fresh weight of shoot (19.8%) and root (24.5%) along with the dry weight of shoot (28.7%) and root (20.2%), as well as the leaf area (28.7%), was observed in the P-1543 cultivar of maize vegetated in K soil.

### 3.2. Physiological Traits of Maize Plant

The chromium toxicity influenced photosynthetic pigments and gaseous exchange measurement of all the maize cultivars differently in both K and S soils as presented in Table 4. In the control treatment, the harmful effects of Cr critically altered the SPAD chlorophyll content (8.5 mg cm^−2^), chlorophyll “a” (0.14 mg g^−1^) and “b” (0.12 mg g^−1^), A rate (5.5 µmol m^−2^ s^−1^), E rate (0.93 mmol m^−2^ s^−1^), gs (44 mmol m^−2^ s^−1^), and total carotenoids content (0.18 mg g^−1^) of maize cultivar FH-985 in S soil. Biochar (3%) application significantly improved the physiological traits in both soils and most distantly increased the SPAD chlorophyll content (21.5%), chlorophyll “a” (21.4%), chlorophyll “b” (19.8%), total carotenoids content (18.8%), A rate (20.3%), E rate (24.8%), and gs (19.2%) of maize cultivar P-1543 in K soil.

**Figure 1:**
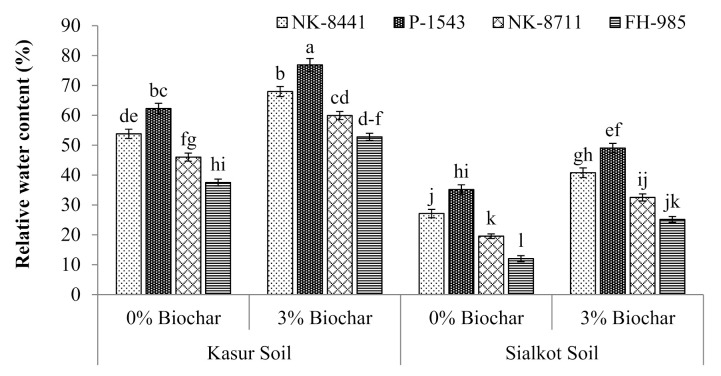
Relative water content of studied maize cultivars with the application of biochar in the tannery polluted soils of Kasur and Sialkot. Mean values are indicated by the columns and the standard error of the mean is shown with bars. All means followed by different letters are significantly different (*p* < 0.05) according to Tukey’s HSD test.

**Figure 2:**
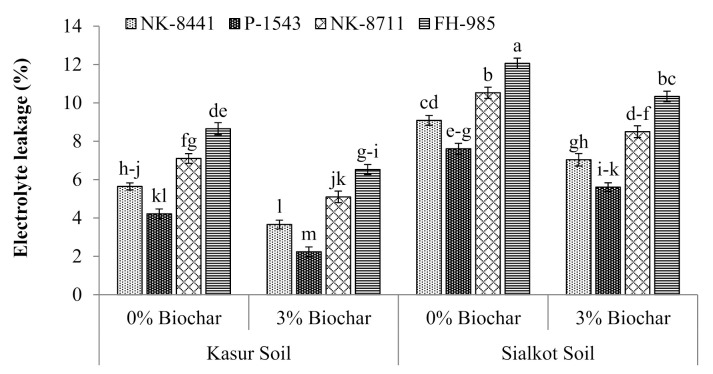
Electrolyte leakage of studied maize cultivars with the application of biochar in the tannery polluted soils of Kasur and Sialkot. Mean values are indicated by columns and the standard error of the mean is shown with bars. All means followed by different letters are significantly different (*p* < 0.05) according to Tukey’s HSD test.

Data showed that the RWC (Figure 1) and EL of all the maize cultivars (NK-8441, P-1543, NK-8711, and FH-985) were also affected by the damaging and toxic effects caused by Cr (Figure 2). A decrease in the RWC and an increase in the level of EL of maize plants were noticed in both soils. In the control treatment, an acute decrease in RWC (12%) and increase in EL (12.1%) of maize cultivar FH-985 was observed in S soil, while treatment involving biochar (3%) application provided relief to all maize cultivars and improved the RWC and EL in both soils. The sharp gain in RWC (23.5%) and decrease of EL (47%) in maize cultivar P-1543 was observed in K soil only.

### 3.3. Biochemical Attributes of the Plants

#### 3.3.1. Stress-Related Metabolites

Rise in stress related metabolites of all the studied maize cultivars (NK-8441, P-1543, NK-8711, and FH-985) was observed in both soils (K and S) as shown in (Table 5). In control treatment, toxic Cr (VI) extremely increased the soluble sugars (83.7 mg g^−1^), lipid peroxidation, and proline content (8.36 μg g^−1^) in the form of malondialdehyde (146 mmol g^−1^) of maize cultivar FH-985 in S soil. A significant reduction in the metabolites activity was found on the application of biochar (3%) in both soils. Biochar strongly decreased the soluble sugars, lipid peroxidation, and proline content in maize cultivar P-1543 up to 43.9%, 48.4%, and 38%, respectively, in K soil.

#### 3.3.2. Measurement of Antioxidant Enzyme Activities

Chromium-induced toxicity in tannery polluted soils also transformed the antioxidant enzyme activities of maize plant (Table 5). An increase in enzymes (APX, GPX, CAT, and SOD) activity of all the studied maize cultivars was found in both soils. Chromium predominantly increased the APX (76.8 nmol min^−1^ mg^−1^), GPX (98.3 nmol min^−1^ mg^−1^), CAT (27.8 nmol min^−1^ mg^−1^), and SOD (289 nmol min^−1^ mg^−1^) of maize cultivar FH-985 in the control treatment of S soil. Biochar (3%) application significantly reduced the antioxidant enzymes activity and principally decreased the activity of APX (50.6%), GPX (58.4%), CAT (52.5%), and SOD (50.3%) of P-1543 maize cultivar in K soil.

### 3.4. Chromium Concentration in Soil and Plant Tissues

The accumulation of Cr greatly varied in the soil as well as in the root and shoot of all the studied maize cultivars (Table 6). Cultivar FH-985 in both soils accumulated Cr (III and VI) mostly in the plant root and shoot tissues and less amounts in the soil. The lowest amount of Cr (III) and Cr (VI) was 18.2 μg g^−1^ and 4.3 μg g^−^^1^, respectively, in K soil. The highest accumulation of Cr (III) was 307 μg g^−1^ in root and 251 μg g^−1^ in shoot, while Cr (VI) was 142 μg g^−1^ in root and 110 μg g^−1^ in shoot of S soil. In contrast to cultivar FH-985, cultivar P-1543 mostly accumulated dominant species of Cr (III and VI) in soil, while it decreased its accumulation in maize roots and shoots. The application of biochar (3%) resolved this issue, by significantly lowering the Cr (VI) concentration (available fraction) and increasing the Cr (III) concentration (less soluble fraction) in soil along with a reduction in Cr (III and VI) accumulation in root and shoot. Moreover, applied biochar increased Cr (III) accumulation up to 67.4 μg g^−1^ in cultivar P-1543 of S soil and decreased the accumulation of Cr (III) (53 and 41 μg g^−1^) and Cr (VI) (27 and 21 μg g^−1^) in root and shoot of K soil, respectively.

### 3.5. Correlation and Principal Component Analyses

**Figure 3:**
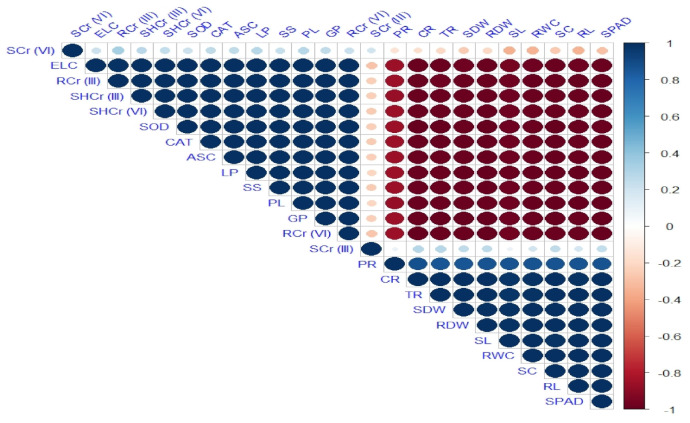
Correlation matrix representing correlation among different attributes of maize crop followed by different treatments, such as Kasur soils (0% biochar) (1) NK−8441, (2) P−1543, (3) NK−8711, (4) FH−985; (3% biochar) (5) NK−8441, (6) P−1543, (7) NK−8711, (8) FH−985; Sialkot soils (0% biochar) (9) NK−8441, (10) P−1543, (11) NK−8711, (12) FH−985; (3% biochar) (13) NK−8441, (14) P−1543, (15) NK−8711, (16) FH−985. Positive correlations are displayed in blue and negative correlations in red. The color legend on the right-hand side of the correlogram shows the correlation coefficients and the corresponding colors. The abbreviations are as follows: Shoot length: SL, Root length: RL, Shoot dry weight: SDW, Root dry weight: RDW, Chlorophyll contents: SPAD, Photosynthetic rate: PR, Transpiration rate: TR, Stomatal conductance: SC, Lipid peroxidation: LP, Proline contents: PL, Total carotenoids: CR, Soil Cr lll: SCrlll, Shoot Cr III: SHCrIII, Root Cr III: RCrIII, Soil Cr VI: SCrVl, Shoot Cr VI: SHCrVI, Root Cr VI: RCrVI, Relative water contents: RWC, Electrolyte leakage: ELC, Catalase: CAT, Ascorbate peroxidase: ASC, Glutathione peroxidase: GP, Soluble sugars: SS, and Superoxide dismutase: SOD.

**Figure 4:**
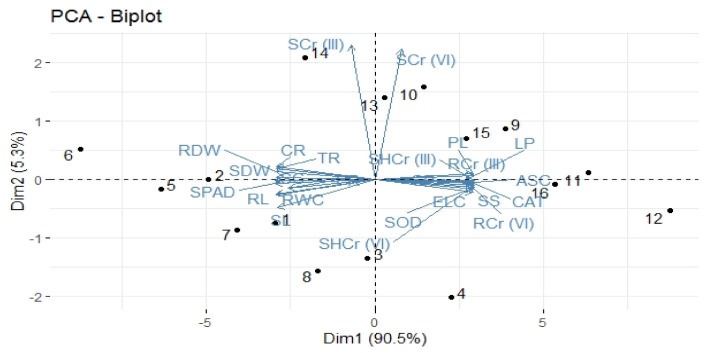
Principal component analysis (PCA) showing biplot (score and loading) of different attributes of maize cultivars. Score plot represents separation of treatments, such as Kasur soils (0% biochar) (1) NK−8441, (2) P−1543, (3) NK−8711, (4) FH−985; (3% biochar) (5) NK−8441, (6) P−1543, (7) NK−8711, (8) FH−985; Sialkot soils (0% biochar) (9) NK−8441, (10) P−1543, (11) NK−8711, (12) FH−985; (3% biochar) (13) NK−8441, (14) P−1543, (15) NK−8711, (16) FH−985. Loading plot shows the loading of each studied variable (arrows) and the arrow length approximates their variance, whereas the angles between them represent their correlation. The abbreviations are as follows: Shoot length: SL, Root length: RL, Shoot dry weight: SDW, Root dry weight: RDW, Chlorophyll contents: SPAD, Photosynthetic rate: PR, Transpiration rate: TR, Stomatal conductance: SC, Lipid peroxidation: LP, Proline contents: PL, Total carotenoids: CR, Soil Cr lll: SCrlll, Shoot Cr III: SHCrIII, Root Cr III: RCrIII, Soil Cr VI: SCrVl, Shoot Cr VI: SHCrVI, Root Cr VI: RCrVI, Relative water contents: RWC, Electrolyte leakage: ELC, Catalase: CAT, Ascorbate peroxidase: ASC, Glutathione peroxidase: GP: Soluble sugars: SS, and Superoxide dismutase: SOD.

Significant positive and negative relationships were observed among all the studied morphological and physiological attributes of the maize plant and Cr concentrations in soil and plant tissues (Figure 3). In the same way, a tremendously positive relationship was also observed among all the measured antioxidant enzymes. Principal component analysis was applied to assess the distribution of different treatments carried out on maize cultivars (NK-8441, P-1543, NK-8711, and FH-985) in tested soils as presented by a biplot (Figure 4). From the biplot of PCA, remarkable outcomes were obtained presenting excessive variability among all the studied parameters measured in different maize cultivars, with the first two major constituents interpreting a variability of 95.8%. C Cultivar FH-985 in maize vegetated in S soil showed the maximum coordinate on the biplot of the PCA, indicating that it is the most effective treatment, followed by cultivar NK-8711 in the same soil type. The PCA biplot also revealed that the concentration of Cr species (III and VI)) is highly positively correlated to each other in root and shoot of the plants and antioxidant enzymes, whereas all the morpho and physiological attributes of the maize plants are highly negatively correlated while they are highly positively correlated among themselves.

## 4. Discussion

Soil pollution caused by the utilization of heavy metal in chemical-based manufacturing industries is a growing problem all over the world. In Pakistan, the lack of policies and their improper implementation has dramatically increased the heavy metal pollution from industries [81]. It is an established fact that low organic matter and Cr accumulation may disturb the soil structure and results in reduced crop growth [82]. Biochar is a recalcitrant carbonaceous charred organic amendment that has the potential to improve soil structure, organic matter, water holding capacity, and nutrient retention in the soil [83,84]. Owing to its large surface area, various functional groups and high CEC biochar could act as an adsorbent material for heavy metal clean-up from the contaminated sites [85,86,87].

In the present study, potential of biochar was compared on Cr accumulation, improvement in growth, physiology, and biochemistry of maize cultivars (NK-8441, P-1543, NK-8711, and FH-985) vegetated in soils polluted with tannery waste produced by tannery industrial complex in two districts of Punjab (Kasur and Sialkot), Pakistan. The results of the present study revealed that the Cr toxicity in tannery waste polluted K and S soils have strongly negatively affected the development and growth of maize cultivars. However, the magnitude of toxicity caused by Cr and its effects on maize cultivars varied in both soils. Maize growth was more affected in S soil as related to K soil. This effect might be related to greater Cr toxicity of S soil. Similarly, alteration in plant physiological processes due to Cr toxicity was also monitored, which might be involved in the growth and improvement of maize cultivars. This variable response of plants in their various growth and development phases under heavy metal Cr toxicity are in accordance with earlier studies [6,14,17,27]. The reduction in root growth under the influence of Cr could be attributed to inhibited root cell division, elongation, and/or an affected cell cycle [88]. Chromium stress probably has decreased the root surface for soil penetration and plant ability to explore the soil surface for water [89,90]. Exposure of seedling roots with Cr most likely has caused tissue collapse, and consequently, failure to absorb water and nutrients, and ultimately stunted plant growth [17,91]. Moreover, localization of Cr into vacuoles, particularly in parenchyma cells and cell walls of xylem perhaps has affected cell activity and might have lowered the water potential [92,93]. Similarly, reduced plant height and shoot weight might have attributed to reduced root growth and subsequently lowered nutrient and water transport to the aerial part [94]. In addition, Cr translocation from root to shoot might have affected the cellular metabolism of shoots and reduced the shoot growth [95].

Development of leaf area, growth, and total number of leaves aggressively direct the plant growth and its development. The reduction in leaf area could be related to structural abnormalities, chlorosis, and/or tissue necrosis because of Cr toxicity [96]. The addition of biochar gave better results in terms of improved plant growth and physiological attributes. This improvement might be associated to high surface area of biochar that have sorbed most of the Cr and lowered its availability and toxicity for plant growth and development [87,97]. Similar results regarding improvement in plant growth by application of biochar in lead (Pb) contaminated soil were observed by Jiang et al. [98]. A reduction in the available portion of cadmium (Cd), zinc (Zn), and Pb due to biochar (10%) application was reported by Houben et al. [36]. In our results, Cr toxicity have affected the growth of maize cultivars and it was observed in the order P-1543 > NK-8441 > NK-8711 > FH-985. Cultivar P-1543 was less affected in both soils showed the better performance by resistivity against Cr stress, whereas the growth of maize cultivar FH-985 was maximally affected, indicating sensitivity to Cr toxicity. It is well known that biochar application results in the supply of crop nutrients for a longer time, and thus, improve the growth and productivity of crops under stressed conditions [39,99,100].

The positive effects of biochar were also observed in terms of improved plant physiological process. Photosynthetic pigments, i.e., SPAD chlorophyll, chlorophyll “a” and “b” as well as total carotenoids, are molecules involved to capture light energy of definite wavelength essential for photosynthesis and were affected by Cr stress in K and S soil. Impaired activities of enzymes involved in the biosynthesis of pigments and reduction in uptake of magnesium (Mg) and nitrogen (N) due to Cr toxicity could be a reason of reduced photosynthetic pigments [101,102]. Moreover, it might also be associated with the increased production of ROS that results in lowering the photosynthetic capacity of the plants under Cr stress [103]. Chromium accumulation might also have reduced the energy utilization, which in turn reduced the activity of photosynthetic pigments [104,105]. Similarly, the plant photosynthetic rate (A) in terms of the CO_2_ fixation rate was significantly decreased by Cr toxicity of K and S soil. Harmful influence of Cr probably has affected the activities of carbon fixation enzymes and electron transport chain [106]. Reduced CO_2_ assimilation might also be related to low efficiency in excitation capture and PS-II quantum yield [107,108]. Chromium-induced abnormalities also affected the plant transpiration rate (E) with respect to water loss from plant surfaces. The reduction in the transpiration rate may possibly be attributed to reduced water potential and enhanced diffusive resistance [109]. Similarly, stomata, having special cells known as guard cells, control its opening and closing [110]. These stomatal openings endure gas exchange at the cost of water loss and are affected by toxic Cr influence [108]. Our results are in accordance with previous reports [15,91], in which reduced physiological and photosynthetic ability of plants grown under Cr stress was also observed.

The bioaccumulation of toxic Cr metal might have reduced the cell osmotic potential or reduced nutrient uptake and caused an imbalance of the stomatal activity, and therefore, affected plant photosynthetic process and reduced plant growth [111]. In the photosynthetic pigments, Cr toxicity might also have decreased the size of the peripheral part of the antenna complex, degradation or destabilization of the proteins of the peripheral part, or inactivation of enzymes that could lead to decline in chlorophyll content [112,113]. Reduced synthesis of chlorophyll, ultra-structure of chloroplast disorganization, inhibition of electron transport processes, and/or decreased enzymatic activity of Calvin cycle by Cr stress [17] could be possible reasons of the decreased plant photosynthetic process in the present study. In photochemical process, the synthesis of electron may not be efficiently utilized for carbon assimilation; deviation of electrons of PS-I to Cr (VI) in the electron donating side [13,106] could also be a reason of reduced photosynthetic rate, as was evidenced in our results for Cr toxicity. Chromium-induced abnormalities in the ultrastructure of chloroplast could be caused by a poorly created lamellar system with broadly separated thylakoid and little grana [114]. The decrease in relative water content may be due to the reduced root surface and area that might have reduced the plant potential to absorb the water from soil surface [115]. In addition, the Cr toxicity in the plant might also have reduced the longitudinal water movement by decreasing the diameter of treachery vessel [17]. Similarly, ROS are generated as a normal product of plant cellular metabolism. These consist of free radicals such as hydroxyl radical (^•^OH) and superoxide anion (O_2_^•−^) in addition to non-radical molecules such as singlet oxygen (^1^O_2_), hydrogen peroxide (H_2_O_2_), and so forth [116,117]. Chromium stress in plants leads to production of excessive ROS due to cellular homeostasis disruption [118]. Excessive formation of ROS causes oxidative damage in plants, including cell membrane damage and leakage of cell electrolytes [14]. Moreover, enhanced EL in this study might be associated to excessive ROS formation by Cr stress. However, the application of biochar resulted in increased RWC and decreased EL, which might be associated to enhanced availability of essential plant nutrients and improved water holding capacity [32,52,119]. The increased RWC and decreased EL, thus, helped to better sustain maize cultivars under Cr stressed environments [15,120].

Stress-related metabolites, such as the accumulation of soluble sugars, lipid peroxidation, proline, etc., may play a role in inducing resistance to stress by avoiding cell damage against attacks by free radicals [121,122]. An increased content of these metabolites in plants have been reported by environmental stresses such as salinity, drought, temperature, and heavy metals toxicity [123]. The accumulation of proline has multiple functions, such as osmotic adjustment, enzyme protection, detoxification of injurious ROS, and maintaining protein synthesis [124,125]. In the present study, an increased level of proline content has been observed, which might be associated to a plant’s adopted strategies to survive with Cr toxicity [113]. In the same way, an increased level of soluble sugars in plants has also been indicated in various stress conditions [126]. In this study, the accumulation of soluble sugars that was monitored may be due to the increased concentration of Cr in the plant tissues. The accumulation of soluble sugars in the stress form protects and repairs the biomolecules and cell membranes [125]. In addition, soluble sugars in the stress environment could help to retain cell osmotic potential and maintains carbohydrate supplement [122]. Similarly, lipid peroxidation is a chain reaction and is formed by oxidative damage of ROS, which affects cellular membranes, lipoproteins, and other molecules that contain lipids [127]. Here, the increased level of lipid peroxidation in the form of MDA content might be associated to the oxidative damage of Cr.

The detrimental effects of Cr also altered the extent of antioxidant enzymes activities in both soils. Undeniably, the enhancement in activities of APX, GPX, CAT, and SOD enzymes of maize leaves was observed under Cr-polluted soils, which suggests its role in the detoxification of ROS. These results were supported by previous findings [15,100,128]. Susceptible genotypes (FH-985) showed more increase in antioxidants activity compared to tolerant genotypes (P-1543). Similar findings were previously reported by Foroozesh [129] on antioxidant enzyme activity in different bean genotypes against Cd stress. Increased activity of APX might play a function in scavenging of H_2_O_2_ and converting it into water by using ascorbate as an electron donor [118,130]. Enhanced activity of GPX might have protected the protein and lipid membrane against denaturation caused by Cr exposure [15,131]. Similarly, higher activity of CAT lessened the oxidative effect of Cr metal to stromal and thylakoid of chloroplast function [106,132]. Elevated activity of SOD most likely formed primary resistance against oxidative damage and altered O_2_^-^ to H_2_O_2_, which subsequently further transformed it into water by APX and CAT [15,113].

Chromium (VI) and (III) accumulation in maize cultivars were also quite different in both soils. Basic analysis of soils (Table 2) revealed that S soil has higher Cr concentration, and consequently, accumulated more Cr in maize plant (root and shoot). The highest accumulation of Cr was found in cultivar FH-985, which might be associated to its sensitiveness against stress. Structural resemblance of Cr with some essential elements can displace plant mineral nutrient uptake and translocation in a complicated way. The decrease in nutrient uptake in plants could also be due to reduced root growth and its surface [133,134]. Chromium (III and VI) accumulation in plant usually takes place by different mechanisms. In the tannery industry, primarily Cr (III) is released through waste effluent and converted into Cr (VI) under oxidized conditions [15,30,135]. Higher accumulation of Cr (III) in plants, as was observed in our results, could be associated to higher Cr (III) concentration in soil compared to Cr (VI). Moreover, Cr (III) is most likely passively taken up in plants by simple diffusion, whereas Cr (VI) is usually actively taken up in plants by metabolically driven processes consisting of carriers of essential anions such as sulfate (SO_4_^-^) [27,136]. Furthermore, reduction of Cr (VI) into Cr (III) in the plant could also be a reason of greater Cr (III) concentration. Most of the Cr accumulated in plant roots and a little fraction is transported to the aerial part [106], as was observed in the present study. A low concentration of Cr in shoot compared to root could be due to complexation, sequestration, or compartmentalization in the cytoplasm and vacuoles of the root cells [137,138]. Chromium also attached to exchange receptors and became immobile in the roots. The least mobility or poor translocation of Cr in root caused greater accumulation of Cr in root cells [112]. A contradiction of Cr uptake and translocation in the plants are also present. Some authors stated that Cr is reduced from hexavalent form to trivalent form in the plant [13,139,140], while others reported that Cr (VI) is directly taken up and translocated by plants without its reduction [17,137]. Moreover, the reduction of Cr (VI) into Cr (III) in the plants led to the generation of ROS, which also affected the growth of the plant.

The application of biochar significantly reduced the Cr (VI) (available fraction) concentration and increased the Cr (III) concentration in soil, which consequently reduced the accumulation of Cr in aerial part. The improved effect of biochar could be attributed to a reduced Cr uptake in a plant by adsorption on its surface, precipitation, or complex formation leading to enhanced plant growth [32,84,141]. The surface of biochar is usually negatively charged, but it also has positive surface charges [142,143]. The enhanced effect by the addition of biochar could be associated to sorption of Cr (III and VI) on the charged surface of biochar through electrostatic attractions [12,144]. The mineral component of biochar, i.e., oxidates, phosphates, or carbonates, are most likely involved in the formation of various precipitates of Cr, and therefore, might have reduced their bioavailability [83,85]. Oxygen- and hydrogen-containing functional groups present on the surface of biochar probably have interacted with Cr and might have formed complexation and lowered its availability to the plant body [32,84]. Moreover, biochar might also have reduced the Cr (VI) into harmless and less soluble/mobile Cr (III), by donating electrons or through adsorption coupled reduction [36,145,146,147]. In conclusion, cultivar P-1543 performed better over other cultivars (NK-8441, NK 8711, and FH-985) by reduced uptake of Cr in the aerial part of plant, which indicated resistance/tolerance against Cr stress.

## 5. Conclusions

Biochar (3%) application significantly improved the physiology, biochemistry, antioxidant activity, and overall growth and health of maize plants along with a significant reduction in the level of Cr (VI and III) accumulation under tannery polluted Kasur and Sialkot soils. The extent of Cr toxicity, accumulation, and its effects on growth of maize cultivars (NK-8441, P-1543, NK-8711, and FH-985) varied in both soils, and cultivar P-1543 performed better in a stressed environment. Biochar application could be a better approach in order to reduce the Cr toxicity level in tannery polluted soils for the enhancement of growth, physio-biochemical characteristics, and production yield of maize plants. In general, biochar application can ameliorate the consequence of soil contamination caused by industrial waste material.

## Figures and Tables

**Table 1:** Physico-chemical characteristics and elemental composition of biochar.

Physicochemical Characteristics	Unit	Biochar (SB)
pH (1:20)		6.49 ± 0.04
Electrical conductivity (EC) (1:20)	dS m^−1^	1.59 ± 0.03
Cation exchange capacity (CEC)	Cmol_c_ kg^−1^	86.90 ± 1.60
Moisture	%	3.36 ± 0.19
Volatile matter	%	17.32 ± 0.64
Ash content	%	21.82 ± 0.44
Fixed carbon	%	57.50 ± 1.50
Conversion efficiency (yield)	%	51.61 ± 0.13
Surface area	m^2^ g^−1^	84.16 ± 2.11
**Nutritional Composition**		
Carbon (C)	%	54.81 ± 0.40
Hydrogen (H)	%	2.56 ± 0.13
Oxygen (O)	%	19.92 ± 1.70
Nitrogen (N)	%	1.89 ± 0.01
Phosphorus (P)	g kg^−1^	3.34 ± 0.62
Potassium (K)	g kg^−1^	2.13 ± 0.48
Calcium (Ca)	g kg^−1^	2.01 ± 0.26
Magnesium (Mg)	g kg^−1^	7.86 ± 1.24
Zinc (Zn)	mg kg^−1^	84.52 ± 4.27
Iron (Fe)	mg kg^−1^	88.36 ± 3.38
Manganese (Mn)	mg kg^−1^	81.54 ± 1.84
Total Chromium (Cr)	µg kg^−1^	0.06 ± 0.03

The values are mean ± standard error (S.E.) (n = 3).

**Table 2:** Physico-chemical characterization of tannery polluted Kasur (K) and Sialkot (S) soils.

Parameters	Unit	K Soil	S Soil
Organic carbon (OC)	g kg^−1^	3.41 ± 0.52	4.16 ± 0.64
Calcium carbonate (CaCO_3_)	%	2.96 ± 0.54	3.23 ± 0.49
Soil texture	-	Silty loam	Silty clay loam
pH	-	7.75 ± 0.81	7.91 ± 0.89
Electrical conductivity (EC)	dS m^−1^	1.469 ± 0.02	1.969 ± 0.01
Cation exchange capacity (CEC)	cmol_c_ kg^−1^	11.56 ± 1.38	15.72 ± 1.42
Total nitrogen (N)	%	0.054 ± 0.01	0.061 ± 0.01
Available phosphorus (P)	mg kg^−1^	5.46 ± 0.67	7.53 ± 0.82
Extractable potassium (K)	mg kg^−1^	94.0 ± 3.38	112.0 ± 4.31
Cr (VI)	mg kg^−1^	12.45 ± 1.02	18.62 ± 1.29
Cr (III)	mg kg^−1^	40.02 ± 2.03	54.94 ± 2.30
Total Cr	mg kg^−1^	52.47 ± 2.14	73.56 ± 2.43

The values are mean ± S.E. (n = 3); K: Kasur soil; S: Sialkot soil.

**Table 3:** Plant growth parameters of studied maize cultivars NK-8441, P-1543, NK-8711, and FH-985 with the application of biochar in tannery polluted Kasur and Sialkot soils.

Soil	Biochar	Maize Variety	Plant Height (cm)		Fresh Weight (g)		Dry Weight (g)		Leaf Area (cm^2^)
			Shoot	Root	Shoot	Root	Shoot	Root	
K soil	0%	NK-8441	43.1 ± 1.09 de	27.3 ± 0.79 de	17.7 ± 0.51 d	9.4 ± 0.27 de	1.83 ± 0.05 de	1.37 ± 0.03 de	81 ± 1.94 e
		P-1543	48.1 ± 0.93 bc	31.6 ± 0.59 bc	20.1 ± 0.54 bc	10.9 ± 0.30 bc	2.13 ± 0.07 c	1.62 ± 0.04 bc	93 ± 2.73 cd
		NK-8711	38.1 ± 0.48 fg	23.3 ± 0.69 fg	15.2 ± 0.41 e	8.0 ± 0.23 f–h	1.52 ± 0.06 fg	1.13 ± 0.04 fg	69 ± 2.33 fg
		FH-985	33.1 ± 0.96 hi	19.0 ± 0.59 hi	12.5 ± 0.45 fg	6.3 ± 0.21 ij	1.20 ± 0.04 h–j	0.90 ± 0.04 h–j	57 ± 1.51 hi
	3%	NK-8441	51.9 ± 0.74 b	34.0 ± 0.86 b	21.0 ± 0.45 b	12.0 ± 0.25 b	2.42 ± 0.05 b	1.72 ± 0.05 b	106 ± 2.00 b
		P-1543	57.9 ± 1.16 a	38.5 ± 0.49 a	24.1 ± 0.65 a	13.6 ± 0.36 a	2.75 ± 0.07 a	1.94 ± 0.06 a	118 ± 3.13 a
		NK-8711	46.5 ± 0.81 cd	29.9 ± 0.39 cd	18.0 ± 0.68 cd	10.5 ± 0.38 cd	2.08 ± 0.05 cd	1.48 ± 0.04 cd	94 ± 2.60 c
		FH-985	41.6 ± 1.15 ef	26.3 ± 0.60 ef	15.0 ± 0.43 e	9.0 ± 0.27 e-g	1.78 ± 0.04 ef	1.24 ± 0.04 e–g	82 ± 2.21 de
S soil	0%	NK-8441	24.7 ± 0.73 kl	13.7 ± 0.71 jk	7.7 ± 0.34 ij	5.4 ± 0.22 jk	1.09 ± 0.04 jk	0.81 ± 0.05 jk	44 ± 2.06 jk
		P-1543	29.9 ± 0.73 ij	17.9 ± 0.79 hi	10.1 ± 0.43 gh	6.9 ± 0.26 hi	1.40 ± 0.06 g–i	1.05 ± 0.03 gi	56 ± 2.08 hi
		NK-8711	19.6 ± 1.20 m	9.9 ± 0.39 l	5.3 ± 0.35 k	3.9 ± 0.21 l	0.81 ± 0.05 l	0.58 ± 0.03 l	32 ± 1.81 l
		FH-985	14.4 ± 0.97 n	6.2 ± 0.52 m	2.9 ± 0.21 l	2.5 ± 0.20 m	0.52 ± 0.03 m	0.34 ± 0.04 m	20 ± 1.79 m
	3%	NK-8441	30.8 ± 0.97 hi	20.3 ± 0.82 gh	11.6 ± 0.50 g	7.8 ± 0.27 gh	1.46 ± 0.05 gh	1.10 ± 0.03 gh	63 ± 2.63 gh
		P-1543	35.5 ± 0.94 gh	24.5 ± 0.52 ef	14.5 ± 0.53 ef	9.2 ± 0.31 d–f	1.78 ± 0.07 ef	1.35 ± 0.05 d–f	75 ± 2.04 ef
		NK-8711	25.7 ± 0.70 jk	16.2 ± 0.60 ij	8.8 ± 0.23 hi	6.3 ± 0.21 ij	1.13 ± 0.05 ij	0.87 ± 0.04 ij	51 ± 1.68 ij
		FH-985	20.3 ± 0.27 lm	12.4 ± 0.49 kl	5.8 ± 0.22 jk	4.9 ± 0.25 kl	0.82 ± 0.04 kl	0.60 ± 0.03 kl	39 ± 1.42 kl

The values are mean ± S.E. (n = 3). Mean values with different alphabetical letters are significantly different (*p* < 0.05) according to Tukey’s HSD test; K: Kasur soil; S: Sialkot soil.

**Table 4:** Plant physiological parameters of maize cultivars NK-8441, P-1543, NK-8711, and FH-985 with the application of biochar in tannery polluted Kasur and Sialkot soils.

Soil	Biochar	Maize Variety	SPAD Chlorophyll	Chlorophyll a	Chlorophyll b	Total Carotenoids	Photosynthetic Rate	Transpiration Rate	Stomatal Conductance
			(mg cm^−2^)	(mg g^−1^)	(mg g^−1^)	(mg g^−1^)	(µmol m^−2^ s^−1^)	(mmol m^−2^ s^−1^)	(mmol m^−2^ s^−1^)
K soil	0%	NK-8441	30.1 ± 0.61 de	0.54 ± 0.02 d–f	0.44 ± 0.01 d–f	0.65 ± 0.02 cd	17.2 ± 0.41 de	2.90 ± 0.06 d	149 ± 3.15 de
		P-1543	34.0 ± 0.83 bc	0.63 ± 0.02 bc	0.52 ± 0.01 bc	0.77 ± 0.02 b	19.8 ± 0.55 bc	3.37 ± 0.09 c	178 ± 4.99 bc
		NK-8711	26.0 ± 0.67 fg	0.44 ± 0.02 gh	0.36 ± 0.01 g	0.54 ± 0.02 e	14.5 ± 0.47 f	2.40 ± 0.06 e	125 ± 4.67 fg
		FH-985	21.7 ± 0.56 hi	0.35 ± 0.02 i-k	0.29 ± 0.01 hi	0.43 ± 0.02 fg	11.9 ± 0.36 gh	2.00 ± 0.06 f	102 ± 3.07 h–j
	3%	NK-8441	37.0 ± 0.79 b	0.66 ± 0.02 b	0.54 ± 0.02 b	0.78 ± 0.02 b	21.1 ± 0.41 b	3.73 ± 0.09 b	183 ± 3.72 b
		P-1543	41.4 ± 0.97 a	0.77 ± 0.02 a	0.63 ± 0.02 a	0.92 ± 0.02 a	23.9 ± 0.61 a	4.20 ± 0.12 a	212 ± 5.31 a
		NK-8711	33.0 ± 0.84 cd	0.57 ± 0.02 cd	0.46 ± 0.01 cd	0.67 ± 0.02 c	18.2 ± 0.55 cd	3.23 ± 0.07 c	163 ± 4.42 cd
		FH-985	28.5 ± 0.72 ef	0.47 ± 0.02 e-g	0.39 ± 0.01 e–g	0.56 ± 0.02 de	15.4 ± 0.38 ef	2.63 ± 0.09 de	141 ± 3.78 ef
S soil	0%	NK-8441	17.7 ± 0.61 jk	0.33 ± 0.02 Jk	0.27 ± 0.01 ij	0.38 ± 0.02 gh	10.4 ± 0.30 hi	1.83 ± 0.07 f	86 ± 3.69 jk
		P-1543	22.0 ± 0.83 hi	0.43 ± 0.02 g–i	0.35 ± 0.02 gh	0.50 ± 0.02 ef	13.2 ± 0.32 fg	2.40 ± 0.06 e	108 ± 3.85 g–i
		NK-8711	13.8 ± 0.59 l	0.23 ± 0.01 l	0.19 ± 0.01 k	0.27 ± 0.02 i	8.0 ± 0.43 j	1.37 ± 0.07 g	66 ± 3.09 k
		FH-985	8.5 ± 0.42 m	0.14 ± 0.01 m	0.12 ± 0.01 l	0.18 ± 0.01 j	5.5 ± 0.42 k	0.93 ± 0.07 h	44 ± 2.09 l
	3%	NK-8441	24.8 ± 0.66 gh	0.46 ± 0.02 fg	0.37 ± 0.01 fg	0.54 ± 0.02 e	14.4 ± 0.25 f	2.43 ± 0.09 e	120 ± 2.89 gh
		P-1543	28.7 ± 0.82 ef	0.56 ± 0.02 c–e	0.45 ± 0.02 de	0.65 ± 0.02 cd	17.1 ± 0.69 de	2.87 ± 0.07 d	143 ± 5.01 d–f
		NK-8711	20.0 ± 0.62 ij	0.36 ± 0.02 h–j	0.29 ± 0.01 hi	0.42 ± 0.02 fg	11.5 ± 0.47 gh	1.93 ± 0.09 f	100 ± 3.58 ij
		FH-985	16.1 ± 0.31 kl	0.27 ± 0.01 kl	0.21 ± 0.01 jk	0.32 ± 0.02 hi	9.0 ± 0.33 ij	1.47 ± 0.09 g	77 ± 2.44 k

The values are mean ± S.E. (n = 3). Mean values with different alphabetical letters are significantly different (*p* < 0.05) according to Tukey’s HSD test; K: Kasur soil; S: Sialkot soil.

**Table 5:** Plant stress-related metabolites and antioxidant activities of studied maize cultivars NK-8441, P-1543, NK-8711, and FH-985 with the application of biochar in tannery polluted Kasur and Sialkot soils.

Soil	Biochar	Maize Variety	Soluble Sugars	Proline Content	Lipid Peroxidation	Ascorbate Peroxidase	Glutathione Peroxidase	Catalase	Superoxide Dismutase
			(mg g^−1^)	(μmol g^−1^)	(mmol g^−1^)	(nmol min^−1^ mg^−1^)	(nmol min^−1^ mg^−1^)	(nmol min^−1^ mg^−1^)	(nmol min^−1^ mg^−1^)
K soil	0%	NK-8441	37.6 ± 1.62 jk	3.65 ± 0.16 ij	69 ± 2.77 hi	34.7 ± 1.30 jk	46.1 ± 2.00 ij	13.5 ± 0.45 hi	132 ± 4.43 gh
		P-1543	28.8 ± 1.57 lm	2.81 ± 0.14 kl	54 ± 1.93 jk	26.0 ± 1.15 l	34.9 ± 1.83 kl	10.3 ± 0.40 jk	94 ± 4.57 ij
		NK-8711	46.2 ± 1.02 g–i	4.71 ± 0.14 gh	85 ± 2.77 fg	43.1 ± 1.53 g–i	57.4 ± 2.29 f–h	16.8 ± 0.57 fg	164 ± 5.18 ef
		FH-985	56.6 ± 1.67 d–f	5.87 ± 0.15 de	104 ± 3.07 c–e	52.4 ± 1.69 d–f	69.1 ± 2.48 c–e	20.3 ± 0.62 de	203 ± 5.48 cd
	3%	NK-8441	24.5 ± 1.66 m	2.59 ± 0.14 l	43 ± 2.30 k	21.4 ± 1.45 l	26.0 ± 1.72 l	8.1 ± 0.48 k	83 ± 4.12 j
		P-1543	16.2 ± 1.23 n	1.74 ± 0.11 m	28 ± 1.54 l	12.8 ± 1.17 m	14.5 ± 1.52 m	4.9 ± 0.34 l	47 ± 3.85 k
		NK-8711	32.9 ± 1.62 kl	3.37 ± 0.15 jk	57 ± 2.77 ij	28.7 ± 1.30 kl	37.4 ± 1.92 jk	11.1 ± 0.51 ij	114 ± 5.03 hi
		FH-985	42.7 ± 1.27 h–j	4.19 ± 0.17 hi	74 ± 2.35 gh	37.2 ± 1.45 h–j	49.7 ± 2.10 g–i	14.6 ± 0.54 gh	147 ± 5.76 fg
S soil	0%	NK-8441	61.6 ± 1.27 cd	6.47 ± 0.12 cd	114 ± 1.73 c	57.8 ± 1.59 cd	74.7 ± 1.91 c	21.6 ± 0.55 cd	215 ± 5.63 c
		P-1543	51.6 ± 2.08 e–g	5.56 ± 0.14 ef	98 ± 3.00 d–f	48.3 ± 1.45 e–g	63.6 ± 1.92 d–f	18.4 ± 0.55 ef	179 ± 5.52 de
		NK-8711	71.3 ± 1.30 b	7.46 ± 0.17 b	130 ±2.88 b	67.3 ± 1.74 b	86.9 ± 2.10 b	24.8 ± 0.58 b	252 ± 6.04 b
		FH-985	83.7 ± 1.91 a	8.36 ± 0.19 a	146 ± 3.20 a	76.8 ± 2.03 a	98.3 ± 2.50 a	27.8 ± 0.71 a	289 ± 6.70 a
	3%	NK-8441	48.7 ± 1.69 f–h	5.07 ± 0.13 fg	91 ± 2.18 ef	44.8 ± 1.38 f–h	59.3 ± 2.19 e–g	17.2 ± 0.49 fg	168 ± 5.23 ef
		P-1543	38.5 ± 1.21 i–k	4.24 ± 0.13 hi	76 ± 2.77 gh	35.2 ± 1.17 i–k	47.7 ± 1.92 h–j	14.0 ± 0.47 h	135 ± 4.39 gh
		NK-8711	57.5 ± 1.42 de	6.02 ± 0.14 de	107 ± 2.70 cd	53.9 ± 1.66 de	71.8 ± 2.21 cd	20.3 ± 0.55 de	207 ± 6.11 c
		FH-985	69.1 ± 1.64 bc	6.97 ± 0.15 bc	129 ± 2.61 b	65.8 ± 1.81 bc	86.6 ± 2.41 b	24.4 ± 0.60 bc	252 ± 5.52 b

The values are mean ± S.E. (n = 3). Mean values with different alphabetical letters are significantly different (*p* < 0.05) according to Tukey’s HSD test; K: Kasur soil; S: Sialkot soil.

**Table 6:** Chromium concentration in soil, root, and shoot of studied maize cultivars NK-8441, P-1543, NK-8711, and FH-985 with the application of biochar in tannery polluted Kasur and Sialkot soils.

Soil	Biochar	Maize Variety	Soil (μg g^−1^)		Root (μg g^−1^)		Shoot (μg g^−1^)	
			Cr (III)	Cr (VI)	Cr (III)	Cr (VI)	Cr (III)	Cr (VI)
K soil	0%	NK-8441	28.4 ± 0.83 m	12.2 ± 0.40 fg	132 ± 5.00 i	65 ± 2.78 hi	105 ± 4.00 ij	50 ± 2.15 jk
		P-1543	33.5 ± 0.93 kl	14.3 ± 0.42 c–e	102 ± 5.07 kl	50 ± 1.56 jk	79 ± 3.40 kl	38 ± 2.08 lm
		NK-8711	23.2 ± 0.81 n	10.0 ± 0.38 h–j	168 ± 5.38 gh	80 ± 3.46 fg	130 ± 5.13 gh	61 ± 1.34 g–i
		FH-985	18.2 ± 0.77 o	7.9 ± 0.35 kl	197 ± 6.83 ef	99 ± 2.60 de	161 ± 4.78 d–f	74 ± 2.23 d–f
	3%	NK-8441	49.7 ± 1.05 ef	8.7 ± 0.32 jk	82 ± 5.35 l	42 ± 2.80 k	66 ± 4.72 l	32 ± 2.17 m
		P-1543	55.2 ± 1.1 cd	10.8 ± 0.38 g–i	53 ± 3.19 m	27 ± 2.69 l	41 ± 2.89 m	21 ± 1.60 n
		NK-8711	44.1 ± 0.96 gh	6.5 ± 0.30 l	112 ± 7.02 jk	56 ± 2.18 ij	92 ± 5.73 jk	43 ± 2.13 kl
		FH-985	39.0 ± 0.86 ij	4.3 ± 0.29 m	141 ± 5.61 hi	73 ± 3.29 gh	120 ± 4.16 g–i	56 ± 1.66 h–j
S soil	0%	NK-8441	40.4 ± 0.93 hi	17.1 ± 0.49 b	233 ± 5.37 cd	103 ± 3.30 cd	185 ± 4.80 cd	81 ± 1.68 cd
		P-1543	45.6 ± 1.04 fg	19.4 ± 0.52 a	204 ± 4.70 e	88 ± 2.74 ef	155 ± 4.95 ef	68 ± 2.72 e–g
		NK-8711	34.9 ± 0.91 jk	14.9 ± 0.46 cd	263 ± 6.58 b	121 ± 2.24 b	214 ± 5.45 b	94 ± 1.73 b
		FH-985	29.6 ± 0.85 lm	12.8 ± 0.43 e–g	307 ± 4.89 a	142 ± 3.24 a	251 ± 4.91 a	110 ± 2.51 a
	3%	NK-8441	62.2 ± 1.09 b	13.6 ± 0.41 d–f	174 ± 4.44 fg	82 ± 2.84 fg	140 ± 4.83 fg	64 ± 2.20 f–h
		P-1543	67.4 ± 1.06 a	15.7 ± 0.43 bc	143 ± 6.24 hi	65 ± 2.07 hi	110 ± 3.48 h–j	51 ± 1.60 i–k
		NK-8711	57.0 ± 0.96 c	11.4 ± 0.37 gh	207 ± 4.99 de	99 ± 2.27 de	166 ± 6.37 de	76 ± 1.89 de
		FH-985	50.7 ± 0.93 de	9.2 ± 0.32 i–k	236 ± 5.65 bc	115 ± 3.35 bc	200 ± 4.65 bc	91 ± 2.15 bc

The values are mean ± S.E. (n = 3). Mean values with different alphabetical letters are significantly different (*p* < 0.05) according to Tukey’s HSD test; K: Kasur soil; S: Sialkot soil.

## Data Availability

The data presented in this study are available on request from the corresponding author.
